# Supplementary material for: ‘Who Else If Not We’. Medical Students’ Perception and Experiences with Volunteering during the COVID-19 Crisis in Poznan, Poland
Source: Int J Environ Res Public Health. 2022 Feb 17;19(4):2314. doi: 10.3390/ijerph19042314 (PMC8871886; doi:10.3390/ijerph19042314)
Supplement: Supplementary file 1 [file ijerph-19-02314-s001.zip › ijerph-1550882-supplementary.pdf]

Supplementary S1 Interview Questionnaire

Date:
Interviewer:

Introduction

This is a questionnaire for the study on students' experiences with volunteering during the COVID-19 pandemic. I would like to elicit your view on the voluntary service during current health care crisis caused by the outbreak and the way it affected your everyday activity. At the same time, I assure you that the results of this interview will be collected anonymously and used solely for scientific purposes.

Gender………………………………………………………………………………………...

Faculty…………………………………………………………………………………………..
Year of study (while being volunteer) ………………………………………...……………..
Time spent on volunteering ……………………………………………………………………..
Tasks performed ………………..……………………………………………………………….
Earlier volunteering ……………………………………………………………………………..

1. **What was your reaction when you heard about the COVID-19 pandemic and the governmental restrictions?**

What were your thought on in?
How did you feel about it?
How did you respond to this crisis?

2. Why did you decide to engage in volunteering?

What were the motivations behind your decision?
Did you consult your decision with anybody?
Were you concerned about anything while making your decision?
Did you experience any type of pressure to volunteer?
Have you ever engaged in voluntary service before?

3. What tasks did you perform during your voluntary service?

What were your responsibilities?
Did you have contact with patients?
Did you have contact with persons infected with COVID-19?

4. What are your experiences with volunteering during the pandemic?

Did you have any idea regarding the volunteering?
Were afraid of anything?
How did you benefit from volunteering? What was the most satisfying aspect?
What challenges did you face during your service? What was the most difficult aspect?

Was it more difficult that you expected?
Do you regret joining the volunteering during the pandemic?

5. How would you rate the organization of students' volunteering?

Did you feel prepared for working as a volunteer?
Did you undertake any type of training before you started your service?
Were you provided with personal protective equipment? Did you feel safe and secure?
Did have access to psychological support when needed?
How would you rate the motivational system for volunteers?

6. What reactions did you face during your voluntary service?

How did your family, relatives, friends, and university colleagues react to your volunteering?
How did the healthcare professionals and patients treat you as a volunteer?
Have you ever experienced any type of discrimination resulting from your volunteering?

7. Are there any other experiences from your volunteering during the COVID-19 pandemic you would like to share?
